# Preparation of Nanopaper for Colorimetric Food Spoilage Indication

**DOI:** 10.3390/polym15143098

**Published:** 2023-07-20

**Authors:** Zainab Al Tamimi, Longyan Chen, Xiaoxu Ji, Gary Vanderlaan, Matthew D. Gacura, Davide Piovesan

**Affiliations:** 1 Biomedical Engineering Program, Gannon University, Erie, PA 16541, USA; altamimi005@gannon.edu (Z.A.T.); ji001@gannon.edu (X.J.); 2 Biology Program, Gannon University, Erie, PA 16541, USA; vanderla002@gannon.edu (G.V.); gacura001@gannon.edu (M.D.G.); 3 MP-Erie-Co, Erie, PA 16501, USA; 4 Center for Manufacturing and Technology, Gannon University, Erie, PA 16541, USA

**Keywords:** nanocellulose, nanofibrillated cellulose, nanopaper, freshness indicator, chicken spoilage, pH-sensitive dye, RGB image analysis

## Abstract

In this study, we are reporting the fabrication of a nanocellulose (NFC) paper-based food indicator for chicken breast spoilage detection by both visual color change observation and smartphone image analysis. The indicator consists of a nanocellulose paper (nanopaper) substrate and a pH-responsive dye, bromocresol green (BCG), that adsorbs on the nanopaper. The nanopaper is prepared through vacuum filtration and high-pressure compression. The nanopaper exhibits good optical transparency and strong mechanical strength. The color change from yellow to blue in the nanopaper indicator corresponding to an increase in the solution pH and chicken breast meat storage data were observed and analyzed, respectively. Further, we were able to use color differences determined by the RGB values from smartphone images to analyze the results, which indicates a simple, sensitive, and readily deployable approach toward the development of future smartphone-based food spoilage tests.

## 1. Introduction

Food packaging is crucial for the vigilant maintenance of food quality and safety during the storage, transportation, and sale of food. Due to the rising demand for food and industrial packaging materials, the world packaging industry is among the largest and fastest-growing commercial sectors. The global food packaging market size was USD 358.3 billion in 2022. It is estimated that the global food packaging market will reach around USD 478.18 billion in 2028, despite the COVID impact in the past three years, at a compound yearly growth rate of 5.1%, according to the report of “Fortune Business Insights” [1]. Despite innovative strides over the past decades, petroleum-based plastics are still the dominant food packaging materials, such as polyethylene terephthalate, polyethylene, polyvinyl chloride, polypropylene, and polystyrene [2]. Both food production and waste streams regarding these industrial plastic polymers leverage considerable environmental burdens, primarily due to the poor degradability of plastic polymers [3]. There has been an increasing interest in the design and fabrication of food packaging materials based on sustainable bio-based polymers, composed of polysaccharides, edible proteins, and natural polymers [4].

On the other hand, the design of traditional food packaging primarily focuses on providing protection of the food product from mechanical damage, light irradiation, undesirable chemical reactions due to exposure to gases (e.g., oxygen), and colonization by pathogenic microorganisms that can produce microbial toxins [5]. However, there is increasing concern about delivering fresh and safe foods to consumers. This has led to the emergence of intelligent food packaging that utilizes smart indicators to monitor food quality across the logistical chain. Intelligent food packaging is the design of monitoring systems that can detect and inform on the condition of food and/or the surrounding environment during transportation and storage of food, providing real-time feedback to producers, retailers, and end consumers [6]. Various technological approaches have been used for intelligent food packaging, such as radio frequency identification (RFID) tags [7], near-field communication tags [8,9], as well as a suite of chemical or biological approaches including integrity indicators, freshness indicators, and time–temperature indicators (TTI) [10,11]. However, one critical limit factor to new food packaging designs is the cost, i.e., the packaging price should be less than 10% of the overall product [12]. Therefore, low-cost freshness indicators that can monitor food spoilage by detecting metabolites from microorganisms through visual color change are particularly attractive.

Freshness indicators rely on pH-sensitive dyes that can change color by reacting with metabolite gases, such as total volatile basic nitrogen (TVBN, e.g., ammonia, dimethylamine, and trimethylamine), CO_2_, and H_2_S [13]. Often, the monitoring of TVBN is critical for meat spoilage. The generation of TVBN is typically caused by the degradation of proteins in meat by microbial metabolic pathways. TVBN has hence been used as an important biochemical marker to monitor chicken meat spoilage [14]. Several groups reported the fabrication of package materials by incorporating one or more pH-responsive dyes, such as bromocresol green (BCG), bromothymol blue, and methyl red [15]. The color change of the pH dyes requires the dissolution of TVBN in water. Therefore, a moderately humid chamber is required inside the food package to maintain the food indicator detection sensitivity [16]. To maintain moisture, highly hydrophilic packaging materials are often proposed for use in indicator preparation to improve the color response of pH-sensitive dye during food spoilage [17]. However, most of the current plastic films such as PE and PET are considerably hydrophobic. Thus, investigating food packaging materials utilizing sustainable biopolymers composed of hydrophilic surfaces may be an overall solution to these problems.

In this study, we are reporting the development of a food freshness indicator that monitors chicken breast meat spoilage using a nanocellulose paper (nanopaper). Nanocellulose is a derivative of cellulose that originates from wood pulp treatments or bacterial synthesis. Nanofibrillated cellulose (NFC) is a material with dimensions of several nm in diameter but 1 to 2 μm in length [17]. The nano-size property and surface chemical functionality make NFC a hydrogel with good transparency. In our previous work, we reported the use of NFC to prepare a film-based NFC paper (nanopaper) [17,18]. Due to its transparency and superior mechanical properties, nanopaper has been demonstrated to be an excellent platform for biosensing applications, which is particularly suitable for colorimetric assay [17]. On the other side, NFC has also been reported to be an excellent food package material due to its good gas and water vapor barrier, hydrophilicity, and excellent thermal-mechanical stability [19]. In this work, we reported a simple way to prepare a nanopaper food indicator by coating BCG dye on nanopaper. We demonstrated that the nanopaper food indicator could be used for monitoring chicken breast spoilage by both naked-eye-based observation and cellphone image-based analysis.

## 2. Materials and Methods

### 2.1. Chemicals and Materials

Some (2,2,6,6-Tetramethylpiperidin-1-yl)oxidanyl (TEMPO)-oxidized NFC (slurry, 0.8 wt% solid) was purchased from the Process Development Center at the University of Maine. Bromocresol green was purchased from Sigma-Aldrich (Milwaukee, WI, USA). Teflon^®^ film made from Teflon^®^ polytetrafluoroethylene (PTFE) discs and polyethylene terephthalate (PET) film were ordered from McMaster-Carr (Elmhurst, IL, USA). Unless otherwise specified, all the other chemicals were purchased from Sigma-Aldrich. Solutions with different pHs were prepared by adjusting the pH of distilled water using a solution of NaOH (1 N) or a HCl solution (1 M).

### 2.2. Nanopaper and Nanopaper Food Indicator Preparation

In a typical experiment, a slurry of TEMPO–Cellulose Nanofibrils (NFC) was dispersed in distilled water at a nanofiber content of 0.1 wt%, and the suspension was stirred extensively for 2 h at 800 rpm to disperse the NFC hydrogel. Fifty grams of the above suspension were then subjected to vacuum filtration using a hydrophilic PVDF filter membrane (EMD Millipore Corporation, Burlington, MA, USA; pore size: 0.45 μm) mounted on a Büchner funnel. After filtration, a wet transparent NFC hydrogel was formed on top of the filter membrane. The gel “cake” (6 cm in diameter) was further baked in an oven operating at 60 °C for 30 min to remove the remaining surface water. To prevent curling of the gel cake during the baking process, the gel on the filter paper was fixed to the Teflon plate using tape. The gel cake with the filter was carefully sandwiched between PET film and Whatman^®^ No. 1 filter paper and then two Teflon boards. Next, the package was placed and dried under pressure (2.6 MPa) at 70 °C or room temperature for 10 min to form a nanopaper. The nascent nanopaper was then peeled off from the filter membrane and kept inside a thick book to prevent surface curling until further use.

Prior to the preparation of the nanopaper indicators, the nanopaper was punched into small circle discs (6 mm in diameter). Then the discs were immersed in a pH-sensitive dye bromocresol green ethanol solution (1%) for 15 min or 30 min. Afterwards, the dyed nanopaper indicator was air-dried for 1 h prior to use.

### 2.3. Food Storage Test

A package of fresh and boneless chicken breast slices of normal pH (5.9–6.0) was purchased from a local Walmart meat department (Erie, PA, USA). The expiry date on the meat package indicated a two-week window for storage at 4 °C. The chicken breast was replaced in a polypropylene (PP) tray. The as-prepared nanopaper food indicator discs were placed in a plastic weigh cup. The cup with the discs was further placed in the PP tray together with the chicken breast, and the whole package was wrapped and sealed using plastic Glad^®^ Cling Wrap (Clorox Canada, Brampton, ON, Canada). Some space was left between the cup and the plastic wrap to ensure that air inside the package could pass through the food indicator discs. The overall sealed chicken breast package was stored at room temperature (20 °C) for a week. Color changes in the nanopaper indicators were monitored daily using a smartphone (iPhone XR; Apple Inc., Cupertino, CA, USA).

### 2.4. Characterization and Data Analysis

The morphology of the nanopaper was characterized with a scanning electron microscope (SEM, Hitachi S-3000N, Hitachi Ltd., Tokyo, Japan, operating under 12 kV) after being sputter-coated with gold.

Transmittance of the nanopaper was obtained using a UV-vis spectrometer (SpectraMax M5; Molecular Devices, Sunnyvale, CA, USA). A tensile test was conducted to determine the mechanical strength of the nanopaper with a universal testing machine (MTEST-Quattro; ADMET, Norwood, MA, USA; the loading cell is 1 kN). A rectangular strip (5 mm × 50 mm) was prepared for the tensile test. The strain rate was fixed at 20% min^−1^. Tensile strain (ε) was defined as length change (Δl) divided by the original length (l_0_) of the sample.

Digital color images of the indicators incubated in the solutions of different pHs or exposed to the packaged chicken breasts were captured using a smartphone (iPhone XR; Apple Inc., Cupertino, CA, USA). The images were analyzed using ImageJ software Version 1.53t (National Institutes of Health, Bethesda, MD, USA) and the average RGB pixel intensities were collected. All RGB values are the mean values from three captured images of the same indicator. The RGB values of the nanopaper food indicators were normalized using the following equation to those of white printing paper to reduce potential errors caused by lighting, position, and camera angle [20].

R′_x_ = R_x_/R_wb_ (1)

G′_x_ = G_x_/G_wb_ (2)

B′_x_ = B_x_/B_wb_ (3)

where x is the number of storage days; R′_x_, G′_x_, and B′_x_ are normalized values; and R_x_, G_x_, and B_x_ are the original values from the images, respectively. R_wb_, G_wb_, and B_wb_ represent the white background values. Lastly, the color difference in the nanopaper indicator during the chicken breast storage period was calculated through the following formula [21]:

Color difference (RGB) = [(R′_x_ − R′_0_)^2^ + (G′_x_ − G′_0_)^2^ + (B′_x_ − B′_0_)^2^]^1/2^ (4)

where R′_0_, G′_0_, and B′_0_ are the normalized RGB values from day 0, respectively.

All the above data were further analyzed and plotted with Microsoft Excel version 16.69.1.

## 3. Results and Discussion

### 3.1. Nanopaper Preparation

**Figure 1:**
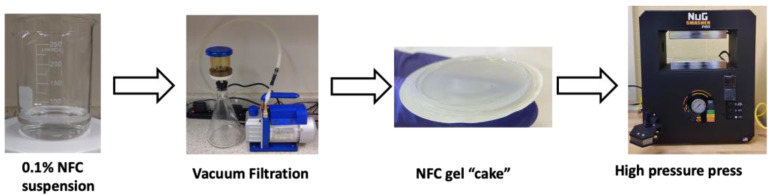
Scheme of the preparation of nanopaper.

Figure 1 shows a scheme of the preparation of nanocellulose paper (nanopaper) through a vacuum filtration procedure, followed by a heating compression step, with slight modification from our previous report [18]. TEMPO-oxidated nanocellulose hydrogel was used to improve the transparency of the nanopaper [22] and provide a high amount of carboxylate groups (about 1.5 mmol/g of the carboxylate content) to bind with ammonium gas released from food during the spoilage process.

**Figure 2:**
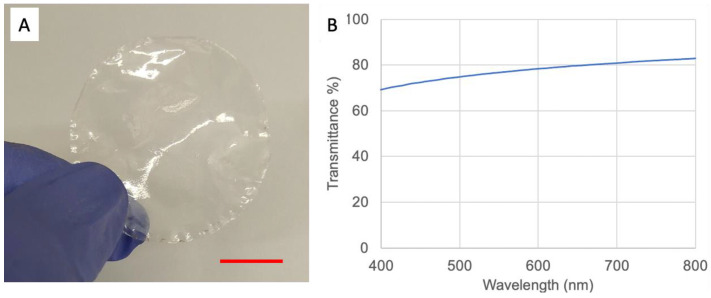
Characterization of nanopaper: (**A**) photograph of nanopaper (red bar, 2 cm); (**B**) transmittance data of the nanopaper in (**A**).

Figure 2A shows that the nascent nanopaper exhibits a transparent plastic-like film. As transparency is important for the later colorimetric-based food indicator, the temperature for heat compression was kept at a low level to prevent decarbonization of anhydroglucuronate units. Following the below discussion on indicator preparation optimizing, we performed the compression at room temperature. Figure 2B shows that the transmittance of the as-made nanopaper reaches more than 70%, from 400 nm to 800 nm, slightly lower than our previous study with compression at 85 °C [17].

### 3.2. Nanopaper Characterization

**Figure 3:**
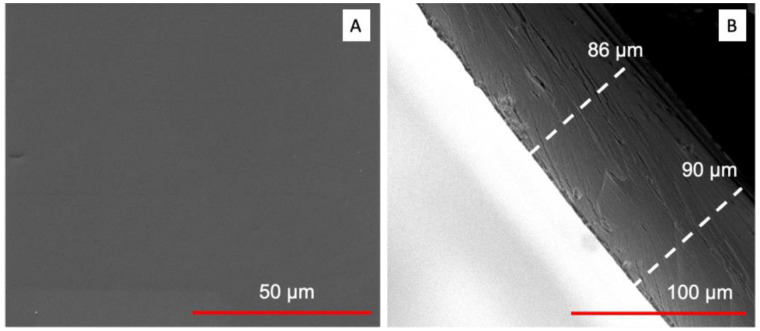
SEM characterization of nanopaper: (**A**) nanopaper surface; (**B**) cross-section view of nanopaper.

We further characterized the nanopaper using a scanning electron microscope (SEM). As shown in Figure 3A, we could observe that the nanopaper exhibited a very flat surface. It should be noted that the thickness can be further adjusted by changing the weight of nanocellulose content used in the filtration step. However, it may take a long time to filter the NFC hydrogel if the nanocellulose content is increased. In our study, we prepared nanopaper with a thickness of around 80 to 90 µm (Figure 2B), which is comparable to the thickness of commercial food packaging film.

**Figure 4:**
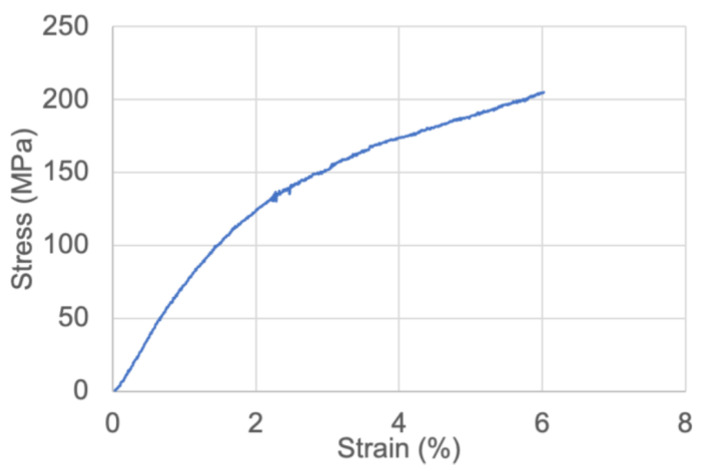
Mechanical testing of nanopaper.

Previous studies indicated that nanopaper has strong mechanical properties [23]. Figure 4 also shows the mechanical testing results for our synthesized nanopaper in this study. The mechanical strength was measured at 200 MPa and Young’s modulus was determined at 7.69 GPa, respectively, despite the low-temperature pressing we used in the study. The mechanical result is comparable and even stronger than some common plastic-based food packaging films such as PET films [24]. Owing to its transparency and excellent mechanical properties, it is expected that the nanopaper formulated in this study would be an ideal component for food packaging. Nonetheless, in this work, we focused primarily on the utility of our nanopaper as a food indicator matrix. As the nanopaper material is useful for both food packaging and food safety monitoring, it is worth noting that the application of spoilage testing dyes (i.e., using ink-jet printing technology) in strategic spots of the inner side of a nanopaper-based packaging film would be an ideal solution.

### 3.3. Nanopaper Food Indicator Preparation and Optimization

Prior to preparation of the nanopaper food indicator, the nanopaper was punched into small discs (about 6 mm in diameter) using a paper puncher. The nanopaper food indicator was then prepared by immersing the nanopaper discs into an ethanol solution of bromocresol green (BCG) at a concentration of 1%, followed by drying at room temperature. BCG is a pH-sensitive dye that belongs to the triphenylmethane family. It changes color from red/yellow at pH 3.8 to blue at pH 5.4. It has been used to titrate growth mediums to monitor the release of ammonium gas during the growth of microorganisms [25].

**Figure 5:**
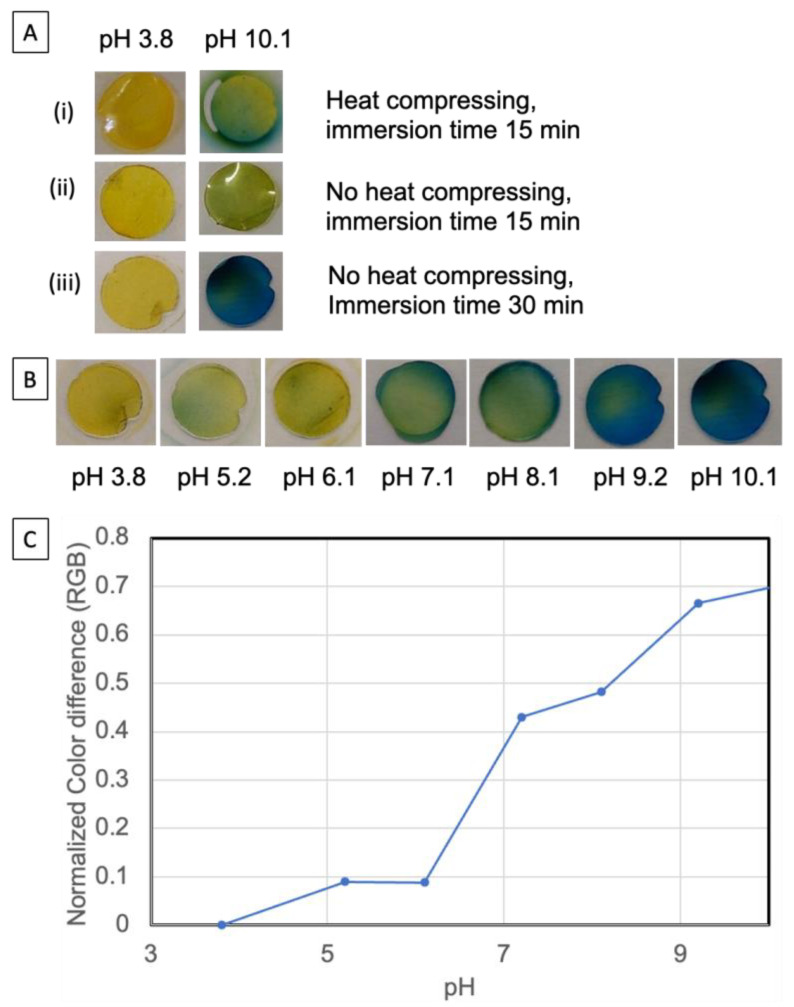
Optimization of nanopaper indicator preparation: (**A**) photos of nanopaper indicator prepared in different conditions; (**B**) the color response of nanopaper in different pHs; (**C**) the color difference (RGB) of the nanopaper indicator for different pHs. The values are normalized based on the RGB value at pH 3.8.

We initially used nanopaper prepared through compression under 70 °C conditions. The dyed nanopaper indicator disc exhibits a yellow color. A higher amount of yellow color rather than the red-orange color from pure BCG could be due to action by surface carboxylate groups from cellulose fibers. Figure 5A shows a yellow-colored sheet under pH 3.8. However, a ring of leaked dye was also observed while adding a drop of solution with pH 10.1 (Figure 5A(ii)). This leakage indicates that the adsorption of the dye on the nanopaper was not very strong. This could be due to higher temperature pressing formulating the nanopaper in an extremely tight manner, leaving only the surface to adsorb the dye. To improve the dye content on the nanopaper, we tested oven drying to remove some of the water content from the NFC filtration “cake”, prior to pressing, and then pressed the hydrogel “cake” at room temperature. It was expected that the nanopaper would retain more water content under these conditions. From Figure 5A(ii), we can see that the intensity of the yellow color increased, and no obvious blue dye leaked at pH 10.1. However, the blue color at pH 10.1 seems very thin. To improve this, we extended the immersion time of the nanopaper into the dye ethanol solution. It was expected that extensive incubation would help the ethanol to penetrate the nanopaper surface with dye and drive more water molecules out of the nanofibrils network. As a result, more dye could be trapped inside the nanofibrils network and hence reduce potential dye leakage. Figure 5A(iii) shows that after 30 min immersion of the nanopaper in the dye solution, the nanopaper indicator did not exhibit dye leakage while exposed to the pH solutions.

We further evaluated the dye under different pH ranges. As shown in Figure 5B, a visual color difference of the nanopaper indicator at various pH values was observed, and the corresponding color-difference-based RGB values are shown in Figure 5C. As we can see, the color changes gradually from yellow to blue with a concomitant increase in pH. It should be noted that the surface of the nanopaper may have gained some impurities during the nanopaper disc manipulation which may affect the pH at local spots. It would be more accurate to observe pH-dependence-based RGB values. In particular, computational techniques now allow a combination of a smartphone digital camera as an acquisition tool and digital-image processing apps to develop a low-cost and more readily available method to measure color.

### 3.4. Evaluation of Nanopaper Food Indictor Response to Chicken Breast Freshness

**Figure 6:**
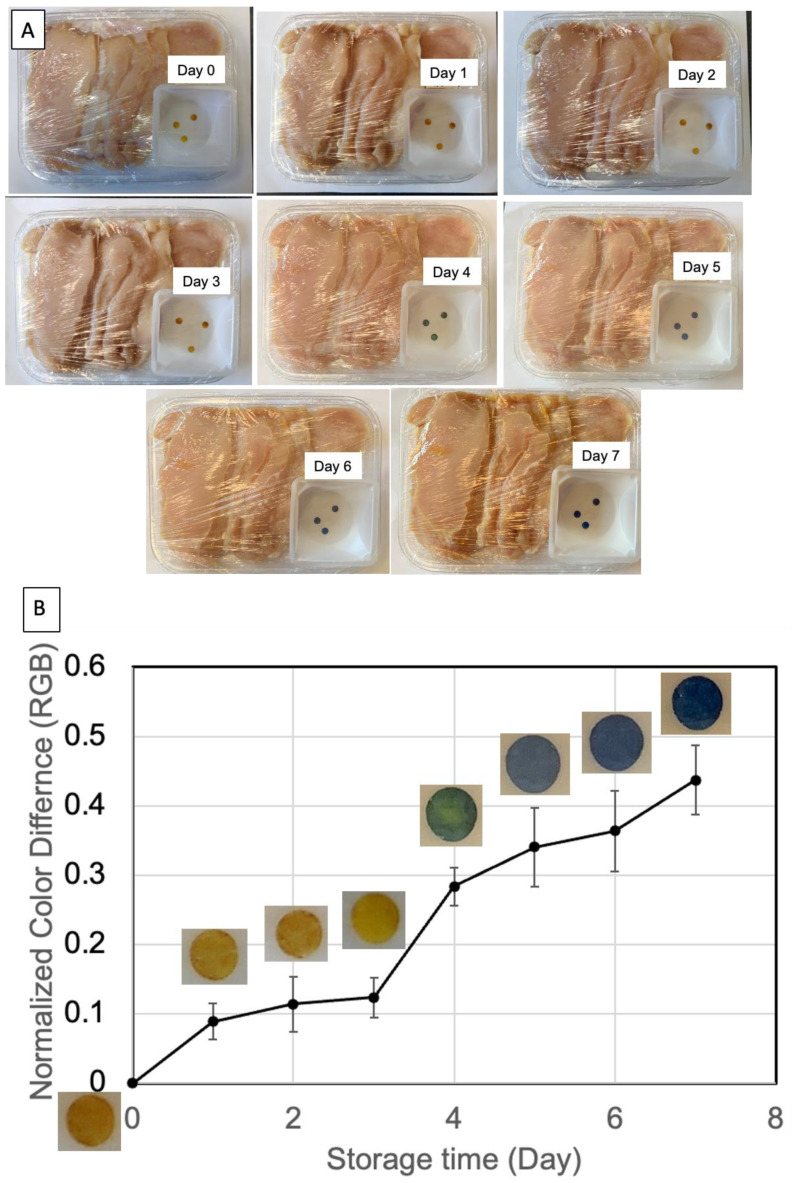
(**A**) Nanopaper indicator for monitoring chicken breast spoilage based on color changes from day 0 to day 7; (**B**) color difference (RGB) in nanopaper indicators used to analyze chicken breast at 20 °C in a week. The values are normalized based on the RGB value at day 1.

To evaluate the nanopaper food indicator response to food freshness, we used fresh chicken breast in our test, as the consumption of chicken consists of one third of the world’s meat consumption [26]. Chicken breast is highly perishable, and freshness can be reduced dramatically over time even when stored in a fridge (at 4 °C). In our study, we kept the food storage temperature at 20 °C for an accelerated spoilage experiment. Figure 6A shows the nanopaper indicator discs that were co-packaged together with the chicken breast without direct contact, and the whole food packaged was wrapped with commercial Glad^®^ Cling Wrap. The color of the nanopaper indicator was initially yellow (i.e., day 0). The yellow color merely turned slightly deeper within the first three days. However, on the fourth day, the color turned blue, suggesting a significantly high amount of TVBN compound produced by the growth of microorganisms. It should be noted that previous studies showed that chicken breast may spoil within seven days at 4 °C, four days at 10 °C, and one day at 20 °C, respectively [27,28]. From the visual color observation, we were not able to see a significant difference in the first three days. This could be due to the good preservation conditions of the fresh meat we received from the grocery store. The package was accidentally moved during the incubation time, which caused the disc positions from day 1 to 3 to be different from the rest. However, there was no leaking observed. We also confirm that the change in color is only for the food indicators in the meat package. Figure S1 shows that there is no significant color change (and neither in RGB values) in the discs kept for a week without meat.

From the color difference obtained using RGB values, we could see that after day 1, there was a significant RGB intensity increase compared to freshly purchased chicken breast. A large RGB increase occurred between days 3 and 4, which correlates with the visual blue color change on the nanopaper indicator. It is difficult to tell if the chicken breast meat spoiled within the first three days in our study, as there is a lack of threshold at this point. We would expect that a systematic study would be required to identify this in our future work. However, the RGB intensity change may indicate that the digital method would be a more sensitive and applicable mode to monitor food spoilage. The current RGB value evaluation is based on manual analysis through ImageJ software. We would expect that more theoretical and advanced studies should be undertaken for analyzing packaged meat quality change and change in nanopaper indicator RGB intensity, as well the development of cellphone apps for easy and readily available food spoilage testing.

## 4. Discussion

In this study, we developed a nanopaper-based food indicator for chicken breast spoilage detection. Spoilage in the meat is due to the degradation of nitrogen-containing compounds, such as proteins, by microbes, causing the accumulation of volatile amines that are termed TVBNs. The detection mechanism thus relies on the determination of TVBN gas using a pH-sensitive dye, BCG. Characterization of microbial species could identify the sources of spoilage and help with food indicator validation, although that is out of the scope of the current study. The dynamics of microbial growth and the dominant species can vary according to the availability of preferred substrate, oxygen, moisture, and the pH of the meat product [29]. For chicken breast, microbes, such as *Pseudomonas* spp., *Shewanella putrefaciens*, and yeast, are commonly present in low quantities during food production and packaging [30,31,32]. Under limited oxygen environments, these microbes change their preferential energy source toward amino acids [33]. Lu et. al. showed that in beef, *Pseudomonas* spp., *Photobacterium* spp., and *Vibrionaceae* spp. contributed to the increase in TVBN levels, resulting in the production of ammonia (NH_3_) and methylamines (MA) [34]. In chicken meat, Lee et. al. found that *Pseudomonas* spp. continuously increased with an increase in storage time, which closely correlates with the amount of TVBN [28]. In a recent study, Saenz-Garcia et. al. showed that *Pseudomonas* spp. has the highest contribution to TVBN formation, comparing *Brochothrix* spp., *Hafnia* spp., *Acinetobacter* spp. during storage at 4 °C [35]. Therefore, we would expect *Pseudomonas* spp. to dominate the contribution of TVBN in our study. In our future study, microbial analysis will be performed to identify the microbe species.

We utilized nanocellulose paper as the substrate to adsorb pH-sensitive BCG dye for a food spoilage indicator. Nanocellulose is a derivative of natural cellulose materials. Cellulose and its derivative forms have shown remarkable properties, including wide availability, inexpensiveness, and degradability. They are also capable of effectively transporting and storing various chemicals. Previous studies have reported the development of filter-paper-based colorimetric food indicators for fish freshness via visual observation [36,37]. In those food indicators, porous filter papers are often used as color-changing layers to absorb dye, while other binding polymers are used to laminate or sandwich to prevent softening during the soaking of moisture inside the filter paper, which subsequently induces color-change distortions [28]. In this study, we used nanocellulose paper (nanopaper). Nanopaper exhibits good mechanical stability (also shown in Figure 4), reducing the risk of surface distortions. Nanopaper also displays a flat 2-D-based surface and has excellent transmittance in visible light, as shown in Figure 2. Our previous study showed that a flat surface provided better signal homogeneity in Raman spectrometry analysis compared to that of porous filter paper, and hence enhanced assay reproducibility [18]. Owing to its strong mechanical properties and being a good gas barrier, as well as being lightweight, nanopaper or nanocellulose film has been reported as an excellent food packaging material [38]. Nanopaper is adapted to printing technology, and thus it is possible that we will be able to print pH-sensitive dye-based (e.g., BCG) patterns on nanopaper. Transparency of nanopaper could enable the visualization of color change in real time.

The cost to produce nanopaper is currently much higher than that of cellulose paper (which is about USD 500 to USD 1500 ton^−1^), which is because of energy- and time-consuming nanocellulose pulp preparation procedures such as chemical treatment of cellulose (USD 2700 ton^−1^ for TEMPO oxidation). However, it is expected that it is possible to reduce the cost by recycling expensive catalyst TEMPO from spent liquid [39]. In our experiment, we used vacuum filtration for small-scale nanopaper preparation, which is also an expensive process. However, large-scale production of nanopaper using a roll-to-roll process has been developed by the VTT Technical Research Centre in Finland, with more efficiency and lower cost [40]. By using a roll-to-roll process, meter-long nanocellulose crystal film was also reported recently [41]. Therefore, nanopaper will potentially become more affordable with technological development and large-scale industrial production.

## 5. Conclusions

In this work, a nanocellulose-based nanopaper food indicator was developed for real-time colorimetric monitoring of chicken breast spoilage, analyzed by both bare-eye observation and using computational RGB analysis from images captured with a smartphone. The nanopaper food indicator consists of a nanocellulose film prepared by vacuum filtration and high-pressure compression with a pH-sensitive dye adsorbed onto the nanocellulose surface. The nanopaper food indicator displayed an optical color change from yellow to blue when the packed chicken was stored for three days at 20 °C. The change of color indicates the growth of microorganisms and release of volatile basic metabolic components. Overall, the nanopaper indicator displayed a distinct color change according to the freshness of food, suggesting that the nanopaper could be a potential platform for intelligent food packaging applications.

## Data Availability

The data presented in this study are available on request from the corresponding authors. The data are not publicly available due to terms and policy.

## Supplementary Materials

The following supporting information can be downloaded at: https://www.mdpi.com/article/10.3390/polym15143098/s1, Figure S1: Comparisons of RGB values between discs kept at room temperature (without meat) between Day 0 and Day 7.
