# Supplementary material for: Preparation of Nanopaper for Colorimetric Food Spoilage Indication
Source: Polymers (Basel). 2023 Jul 20;15(14):3098. doi: 10.3390/polym15143098 (PMC10384993; doi:10.3390/polym15143098)
Supplement: Supplementary file 1 [file polymers-15-03098-s001.zip › polymers-2497309-supplementary.pdf]

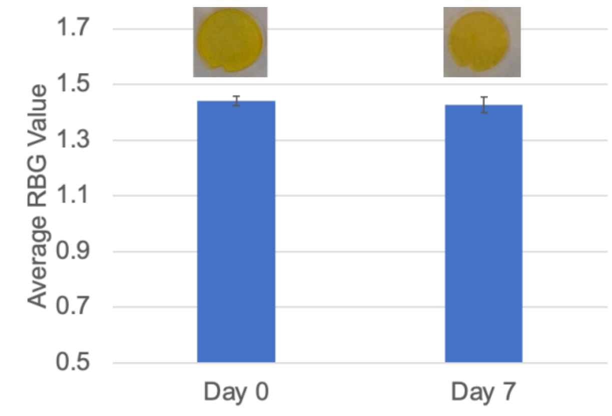

Figure S1. Comparisons of RGB values between discs kept at room temperature (without meat) between Day 0 and Day 7.
